# Identification of NOXA as a pivotal regulator of resistance to CAR T-cell therapy in B-cell malignancies

**DOI:** 10.1038/s41392-022-00915-1

**Published:** 2022-04-04

**Authors:** Xin Yan, Deyun Chen, Yao Wang, Yelei Guo, Chuan Tong, Jianshu Wei, Yajing Zhang, Zhiqiang Wu, Weidong Han

**Affiliations:** 1 School of Medicine, Nankai University, Tianjin, China; 2 Department of Bio-therapeutic, the First Medical Center, Chinese PLA General Hospital, Beijing, China; 3 National Clinical Research Center for Hematologic Diseases, the First Affiliated Hospital of Soochow University, Suzhou, China

**Keywords:** Cancer therapy, Tumour immunology

## Abstract

Despite the remarkable success of chimeric antigen receptor (CAR) T-cell therapy for treating hematologic malignancies, resistance and recurrence still occur, while the markers or mechanisms underlying this resistance remain poorly understood. Here, via an unbiased genome-wide CRISPR/Cas9 screening, we identified loss of NOXA, a B-cell lymphoma 2 (BCL2) family protein in B-cell malignancies, as a pivotal regulator of resistance to CAR T-cell therapy by impairing apoptosis of tumor cells both in vitro and in vivo. Notably, low NOXA expression in tumor samples was correlated with worse survival in a tandem CD19/20 CAR T clinical trial in relapsed/refractory B-cell lymphoma. In contrast, pharmacological augmentation of NOXA expression by histone deacetylase (HDAC) inhibitors dramatically sensitized cancer cells to CAR T cell-mediated clearance in vitro and in vivo. Our work revealed the essentiality of NOXA in resistance to CAR T-cell therapy and suggested NOXA as a predictive marker for response and survival in patients receiving CAR T-cell transfusions. Pharmacological targeting of NOXA might provide an innovative therapeutic strategy to enhance CAR T-cell therapy.

## Introduction

Chimeric antigen receptor (CAR) T-cell therapy has dramatically shifted the landscape of treatment for hematologic malignancies.^1–4^ To date, four US Food and Drug Administration (FDA)-approved CD19-targeted CAR T-cell products have been approved for treating acute lymphoblastic leukemia (ALL), relapsed/refractory (R/R) certain types of large B-cell lymphoma, or chronic lymphocytic leukemia.^5–8^ However, despite these encouraging advancements, ~20% of CD19^+^ R/R B-cell ALL patients who received an infusion of CD19 CAR T cells had no complete remission within 3 months, and approximately 50% of patients relapsed within a year.^9^ Hence, the key challenge is uncovering the underlying resistance mechanisms to CAR T-cell therapy and surmounting these barriers. Mechanisms hampering CAR T-cell therapy are traditionally categorized into two forms: antigen loss or reduction in tumor cells and T-cell dysfunction.^10–15^ Numerous studies, including ours, have attempted to create novel therapeutic strategies to overcome resistance to CAR T-cell therapy by expanding CAR T-cell antigen targets or ameliorating CAR T-cell dysfunction; however, a subset of patients still have no response or eventually relapse,^16–19^ suggesting that the mechanisms responsible for resistance to CAR T-cell therapy are diverse. These two traditional mechanisms of tumor escape and T-cell dysfunction do not fully explain the causes of CAR T-cell failure, suggesting nonantigen-dependent factors in tumor cells might play an important role in resistance to CAR T-cell therapy. Recently, sporadic laboratories began to focus on the effect of nonantigen-dependent factors in tumor cells on the efficacy of CART therapy.^20–22^ However, the underlying intrinsic mechanism of tumor cell resistance to CAR T-cell therapy remains unclear and needs further exploration.

Genome-wide CRISPR/Cas9 screening is a powerful tool extensively used to discover novel targets or molecular resistance mechanisms for cancer therapy.^23^ Genome-wide CRISPR screening in various models has identified that intrinsic regulators of cancer cells mediated the evasion of T cell-mediated killing.^24–26^ Due to the different structures and non-MHC-dependent killing forms, the mechanisms of cancer cell resistance to CAR T cells are not completely identical to those of T cells.^27^ Thus, unbiased CRISPR/Cas9 screening provided us with a favorable way to discover novel mechanisms of tumor cells escaping from CAR T-cell therapy.

In this study, we systematically explored a novel mechanism of resistance to CAR T cells using genome-wide CRISPR/Cas9 screening, and highlighted a central role for NOXA, a B-cell lymphoma 2 (BCL2) family protein, in predicting susceptibility to CAR T-cell therapy in B-cell malignancies. Our work provided evidence for developing mechanism-based combination therapies to improve the efficacy of CAR T-cell therapy in B-cell malignancies.

## Results

### CRISPR screening identifies essential regulators of resistance to CAR T-cell therapy

We performed a genome-scale CRISPR/Cas9 screening in the CD19^+^ human ALL cell line Nalm6 to systematically identify genetic perturbations that regulate resistance to CAR T-cell therapy. We transduced Nalm6 cells with the CRISPR/Cas9 lentivirus Brunello short-guide RNA (sgRNA) library^28^ and then obtained cells under puromycin pressure.

**Fig. 1:**
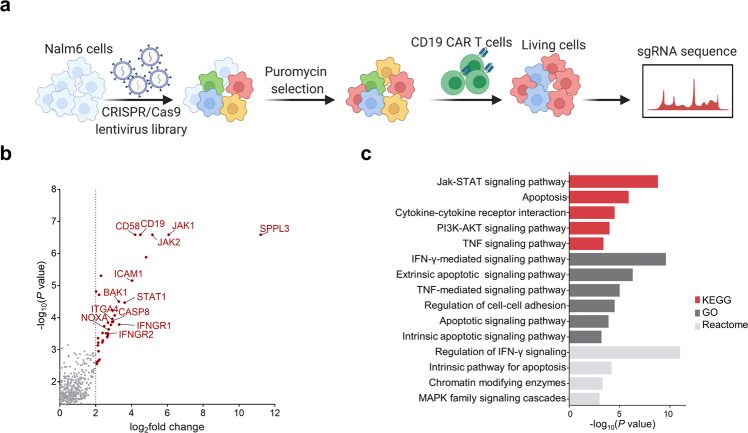
Genome-wide CRISPR/Cas9 screening in Nalm6 cells cocultured with CD19 CAR T cells. **a** Schematic of the CRISPR/Cas9 screening. **b** Volcano plots of essential enriched sgRNAs after screening. **c** KEGG, GO, and Reactome pathway enrichment analysis of genes identified in CRISPR screening

To better reflect the long-term high tumor burden in vivo, tumor cells were repeatedly challenged with CD19 CAR T cells every 3 days for 15 days at an E:T ratio of 1:50. Changes in sgRNA abundance of living cells with or without CD19 CAR T-cell challenges were compared by deep sequencing, and gene analysis was performed using the MAGeCK algorithm^29^ (Fig. 1a and Supplemental Table 1). Screening from CRISPR/Cas9 libraries, we identified significant enrichment of sgRNAs targeting CD19, echoing previous findings that CD19 loss mediated resistance to CAR T cells, thus confirming the reliability of our screening (Fig. 1b). We also observed significant enrichment of sgRNAs targeting SPPL3, which has recently been shown to impede antibody and receptor interactions with HLA class I glycoproteins.^30^ The results suggest that loss of SPPL3 might also be a novel mechanism for tumor cells to escape from CAR T cells (Supplemental Table 1).

Functional Kyoto Encyclopedia of Genes and Genomes (KEGG), Gene Ontology (GO), and Reactome pathway enrichment analyses of genes with significantly enriched sgRNAs suggested that these genes were involved in the regulation of interferon-gamma (IFN-γ) signaling, apoptotic signaling, PI3K-Akt signaling, cell adhesion, and MAPK cascade pathways, which revealed the diversity of mechanisms of resistance to CAR T-cell therapy (Fig. 1c). Among the list of pathways, apoptotic signaling pathways were dramatically enriched in the KEGG, GO, and Reactome databases, implying that apoptotic signaling pathways might be essential regulators in determining the resistance of tumor cells to CAR T cell-mediated killing. CAR T cells lead to the death of target cells via perforin and granzyme-induced mitochondrial-mediated intrinsic apoptosis. Interestingly, sgRNAs targeting NOXA, a BCL2 family protein in the intrinsic apoptotic pathway^31^ were significantly enriched in our CRISPR screening with a log2FC of 2.7233 in Nalm6 cells repeatedly challenged with CD19 CAR T cells (Fig. 1b). Although NOXA silencing or inhibition is a common mechanism to escape targeted therapies and BCL2 inhibitors,^32–34^ the role of NOXA in CAR T-cell therapy is unknown.

### Knockout of NOXA in cancer cells empowers resistance to CAR T-cell therapy

**Fig. 2:**
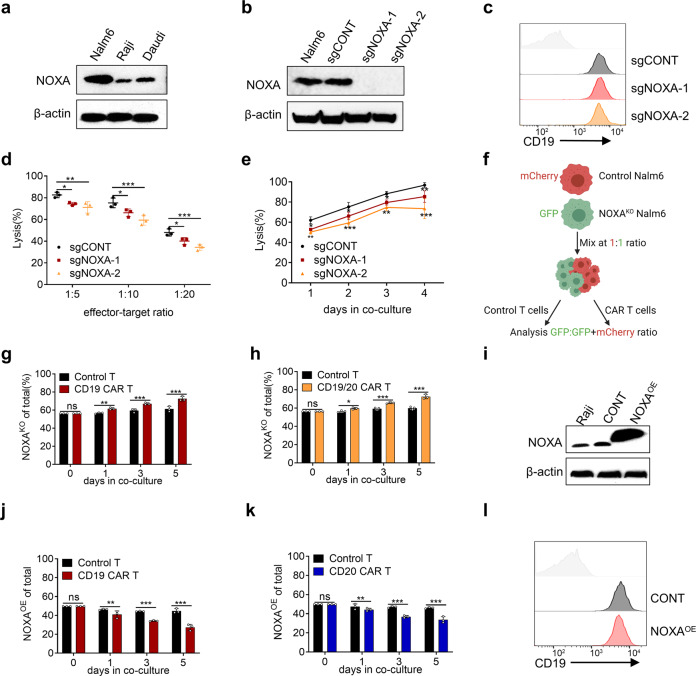
NOXA plays a pivotal role in resistance to CAR T cells. **a** Western blot analysis of NOXA protein levels in CD19^+^ B-lymphoid cell lines. **b** Western blot analysis of NOXA protein levels in the sgCONT and NOXA^KO^ Nalm6 cell lines. **c** Flow cytometry analysis of the level of CD19 expression in the sgCONT and NOXA^KO^ Nalm6 cell lines. **d** Cytotoxic analysis of sgCONT and NOXA^KO^ Nalm6 cells cocultured with CD19 CAR T cells on day 2 at different E:T ratio. **e** Cytotoxic analysis of sgCONT and NOXA^KO^ Nalm6 cells cocultured with CD19 CAR T cells (1:10 E:T ratio) over time. **f** Experimental design of growth competition assay. **g**, **h** mCherry^+^ sgCONT Nalm6 cells were combined with GFP^+^ NOXA^KO^ Nalm6 cells at an ~1:1 ratio and cocultured with CD19 CAR T or tandem CD19/20 CAR T cells. The proportion of GFP^+^ tumor cells over time is shown. **i** Western blot analysis of NOXA protein levels in control and NOXA^OE^ Raji cells. **j**, **k** mCherry^+^ control Raji cells were combined with GFP^+^ NOXA^OE^ Raji cells at an ~1:1 ratio and cocultured with CD19 CAR T or CD20 CAR T cells. The proportion of GFP^+^ tumor cells over time is shown. **l** Flow cytometry analysis of the level of CD19 expression in control and NOXA^OE^ cell lines. Differences among groups were calculated with two-way ANOVA tests. Values are shown as the mean ± SD of triplicates. *P < 0.05, **P < 0.01, and ***P < 0.001

To explore the role of NOXA in resistance to CAR T-cell therapy, we examined NOXA protein levels in CD19^+^ ALL and lymphoma cell lines (Nalm6, Raji, and Daudi, Fig. 2a). We established NOXA stable knockout (NOXA^KO^) cell lines by infecting Nalm6 cells with two lentiviral sgRNAs contained in our library, which were verified by Western blotting (Fig. 2b). As expected, Knockout of NOXA had no effect on the expression of CD19 by flow cytometry (Fig. 2c). In the cytotoxicity assay, NOXA^KO^ cells decreased susceptibility to CD19 CAR T cell-mediated killing (Fig. 2d, e). Next, we conducted a competitive coculture experiment (Fig. 2f). mCherry-labeled sgCONT Nalm6 cells or GFP-labeled NOXA^KO^ Nalm6 cells were cocultured competitively in the presence of control T cells or CD19 CAR T cells, and the relative proportion of NOXA^KO^ Nalm6 cell lines was detected by flow cytometry over time. When cocultured with CD19 CAR T cells, the proportion of NOXA^KO^ Nalm6 cells increased over time compared with that of sgCONT Nalm6 cells (Fig. 2g and Supplemental Fig. 1a, b). Similar results were also shown with tandem CD19/CD20 CAR T cells (Fig. 2h and Supplemental Fig. 1c, d). Conversely, overexpression of NOXA (NOXA^OE^) via lentiviral transduction sensitized Raji cells to CD19 and CD20 CAR T cells (Fig. 2i–k and Supplemental Fig. 1e, f). Moreover, overexpression of NOXA did not affect CD19 expression in Raji cells (Fig. 2l). Together, these data suggest that NOXA plays an important role in the success of CAR T-cell therapy and that the knockout of NOXA in cancer cells empowers resistance to CAR T cells.

### NOXA disruption impairs apoptosis of tumor cells rendering resistance to CAR T cells

**Fig. 3:**
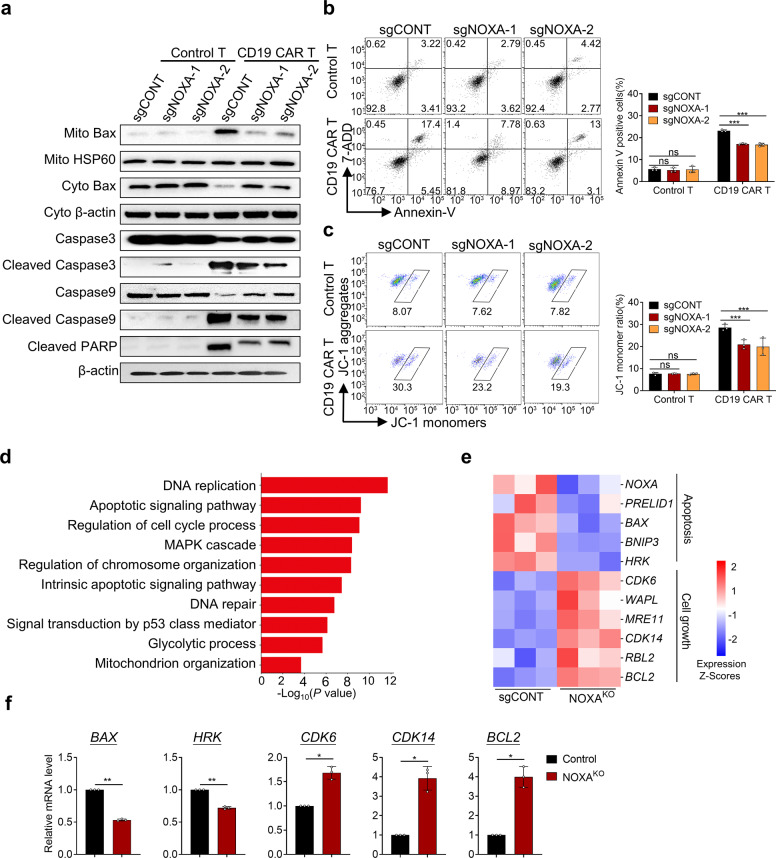
NOXA knockout induces resistance to CAR T cells via the apoptotic pathway. **a** sgCONT and NOXA^KO^ Nalm6 cells were treated with control or CD19 CAR T cells before western blotting assays. Mitochondrial protein levels were normalized to HSP60, and the cytosolic protein and total protein levels were normalized to β-actin. **b**, **c** sgCONT and NOXA^KO^ Nalm6 cells were collected and stained with Annexin V/7-ADD and JC-1 and analyzed by flow cytometry after coculture with control or CD19 CAR T cells. **d** GO enrichment analysis of differentially expressed genes between sgCONT and NOXA^KO^ Nalm6 cells in biological process functions. **e** Heatmap of selected genes with differential expression (*P* < 0.05) related to apoptosis and cell growth between sgCONT and NOXA^KO^ cells after coculture with CD19 CAR T cells. **f** *BAX*, *HRK*, *CDK6*, *CDK14*, and *BCL2* expression was compared between sgCONT and NOXA^KO^ cells after coculture with CD19 CAR T cells by real-time PCR. Differences among groups were calculated with two-way ANOVA tests. Values are shown as the mean ± SD of triplicates. ns: not significant (*P* > 0.05); *P < 0.05, **P < 0.01, and ***P < 0.001

Next, we explored how NOXA disruption rendered resistance to CAR T cells in cancer cells. We identified that NOXA^KO^ Nalm6 cells caused a significant decrease in mitochondria-localized Bax and a corresponding increase in cytosolic Bax when cocultured with CD19 CAR T cells (Fig. 3a). The cleavage of caspase3, 9, and PARP was significantly reduced in NOXA^KO^ Nalm6 cells (Fig. 3a). Moreover, NOXA^KO^ Nalm6 cells exhibited a substantial decrease in the apoptosis and mitochondrial membrane potential after exposure to CD19 CAR T cells (Fig. 3b, c). However, the knockout of NOXA did not change the proliferation and apoptosis of tumor cells without CAR T cells (Supplemental Fig. 2a–c). In addition, knockout of NOXA in tumor cells did not alter CAR T-cell-activated phenotype, as indicated by the markers CD25 and CD69 or the memory phenotype measured by markers CD45RO and CD62 L after 24 h of coculture (Supplemental Fig. 2d, e). To further investigate the molecular mechanism of NOXA loss in tumor cells against CAR T cell-mediated killing, transcriptome differences between NOXA^KO^ Nalm6 cells and Nalm6 cells with control sgRNA in the presence of CAR T cells were analyzed. Transcriptomic analysis showed significant changes in gene expression in NOXA^KO^ tumor cells compared to sgCONT tumor cells under CD19 CAR T-cell pressure (Supplementary Fig. 3a). Pathway enrichment analysis showed that the differentially expressed genes were mainly enriched in DNA replication, apoptotic signaling pathway, cell cycle, and MAPK cascade (Fig. 3d). NOXA^KO^ tumor cells revealed downregulated expression of genes associated with apoptosis, including *PRELIDE*, *BAX*, *BNIP3*, and *HRK*. Cell growth-related genes such as *CDK6*, *WAPL*, *CDK14*, and *BCL2* were also significantly upregulated in NOXA^KO^ tumor cells (Fig. 3e). Consistently with transcriptomic analysis, we also observed marked differences in the mRNA levels of *BAX, HRK, CDK6, CDK14*, and *BCL2* between the sgCONT and NOXA^KO^ tumor cells after co-incubated with CD19 CAR T cells (Fig. 3f).

Taken together, our data indicate that NOXA ablation might impair apoptosis of tumor cells, rendering resistance to CAR T-cell therapy.

### Loss of NOXA in tumor cells exhibits lower susceptibility to CAR T-cell therapy in vivo

**Fig. 4:**
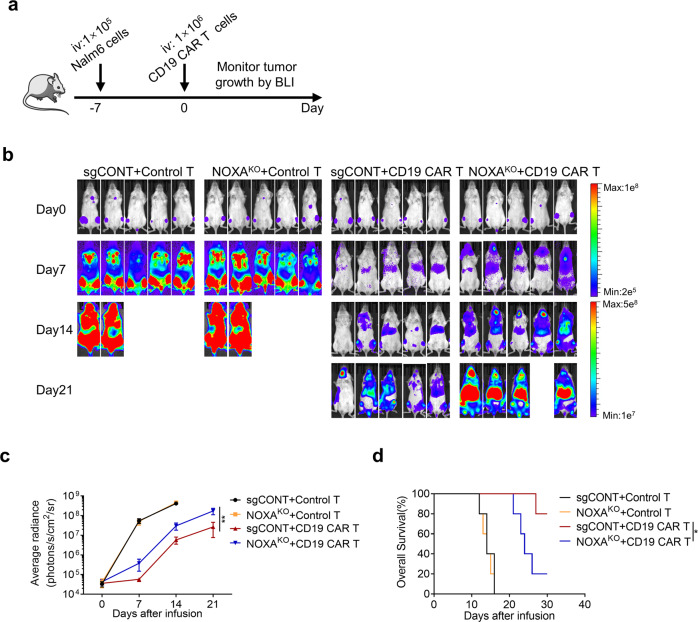
Knockout of NOXA reduces the sensitivity of tumor cells to CAR T-cell therapy in vivo. **a** Experimental timeline comparing the susceptibility of sgCONT Nalm6 and NOXA^KO^ Nalm6 tumor-bearing mice to CD19 CAR T cells. **b** Nalm6 tumor progression as evaluated by bioluminescence imaging (*n* = 5 mice per group). **c** Mouse tumor burden (average radiance). The indicated *P* value was determined by two-way ANOVA test. **d** Survival of mice inoculated with sgCONT or NOXA^KO^ Nalm6 cells and treated with control T cells or CD19 CAR T cells. Log-rank tests were used to determine statistical significance. Values are shown as the mean ± SD of five mice per group. ns: not significant (*P* > 0.05); *P < 0.05, **P < 0.01, and ***P < 0.001

To assess the effect of NOXA expression in tumor cells on the antitumor efficacy of CAR T cells in vivo, we engrafted NOD-Prkdcscid Il2rgnull (NPG) mice with sgCONT or NOXA^KO^ Nalm6 via tail vein, and then infused control T cells or CD19 CAR T cells intravenously (Fig. 4a). Consistent with in vitro findings, CD19 CAR T cells (1e^6^/mouse) had poor ability to eliminate tumors established with NOXA^KO^ Nalm6 cells (Fig. 4b, c). Of note, we also observed a significant reduction in the survival of NOXA^KO^ Nalm6 tumor-bearing mice (Fig. 4d), suggesting that mice xenografted derived from NOXA^KO^ Nalm6 cells exhibited lower susceptibility to CD19 CAR T cells than xenografted mice from sgCONT Nalm6 cells.

### Clinical relevance of NOXA protein levels to CAR T-cell therapy in R/R B-cell lymphoma

**Fig. 5:**
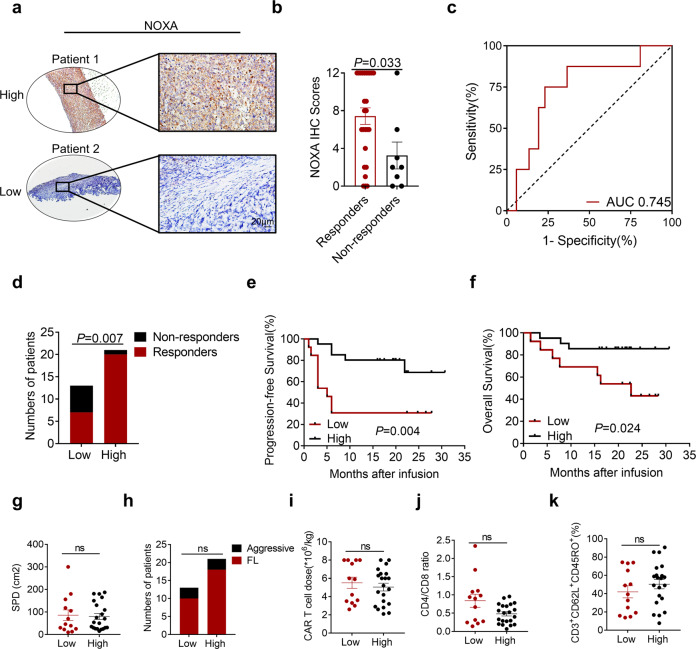
NOXA expression is related to clinical outcomes after CAR T-cell infusion. **a** Representative images of immunohistochemical (IHC) staining for high and low NOXA expression in R/R B-cell lymphoma before treatment with tandem CD19/CD20 CAR T cells. The outlined areas in the left images are magnified on the right. Scale bars, 20 μm. **b** NOXA expression scores by IHC in responders and non-responders. A two-tailed Student’s *t* test was used to analyze the differences between two groups. **c** The ROC curve of NOXA IHC scores in responders and non-responders. **d** The number of responders and non-responders in the low NOXA expression group and high NOXA expression group. A Fisher’s exact test was used for analysis. **e**, **f** Kaplan–Meier plot of progression-free survival and overall survival in the low NOXA expression group and high NOXA expression group. Log-rank tests were used to analyze the significance between the two groups. **g**–**k** Baseline characteristics of patients and transfused CAR T cells between two groups, including tumor burden, lymphoma type, CAR T-cell dose, CD4/CD8 ratio, and memory phenotype Two-tailed Student’s *t* test were used to analyze the differences between two groups. Values are shown as the mean ± SD. ns: not significant (*P* > 0.05); *P < 0.05, **P < 0.01, and ***P < 0.001

Based on our preclinical observations, we next investigated the clinical relevance of NOXA protein levels in patients with R/R B-cell lymphoma to CAR T-cell therapy outcomes. Immunohistochemistry (IHC) was used to examine the NOXA protein levels in all available tumor tissue samples collected prior to tandem CD19/20 CAR T-cell therapy^16,35^ from responders who achieved a complete or partial response (*n* = 27) and from non-responders (*n* = 7). The results showed that the level of NOXA expression was significantly higher in the responders than in the non-responders (*P* = 0.033, Fig. 5a, b). Areas under the ROC curve and 95% CIs were 0.745 (0.55–0.94) for response status within the range of the NOXA IHC scores, indicating the strong distinguishing ability of the NOXA IHC scores (Fig. 5c). We quantified NOXA expression in IHC images and classified samples into low NOXA expression group (IHC scores <4) and high NOXA expression group (IHC scores ≥4).^36^ We identified that patients with low NOXA expression responded worse to CAR T-cell therapy than those with high NOXA expression (*P* = 0.007, Fig. 5d). Notably, a Kaplan–Meier survival analysis of the expression of NOXA and clinical outcomes further showed that a low protein level of NOXA was related to worse progression-free survival (PFS) in this patient cohort (*P* = 0.004, Fig. 5e). A similar trend was shown in the overall survival analysis (*P* = 0.024, Fig. 5f). In addition, it was confirmed that tumor burden, lymphoma type, CAR T-cell dose, CD4/CD8 ratio, and memory phenotype were not significantly distinct between the low and high NOXA expression groups, which ruled out the interference of these factors (Fig. 5g–k). Together, these data provide overwhelming clinical evidence that NOXA might serve as a great prognostic marker in B-cell malignancies.

### Histone deacetylase inhibitors upregulate NOXA expression and enhance tumor cell vulnerability to CAR T cells

**Fig. 6:**
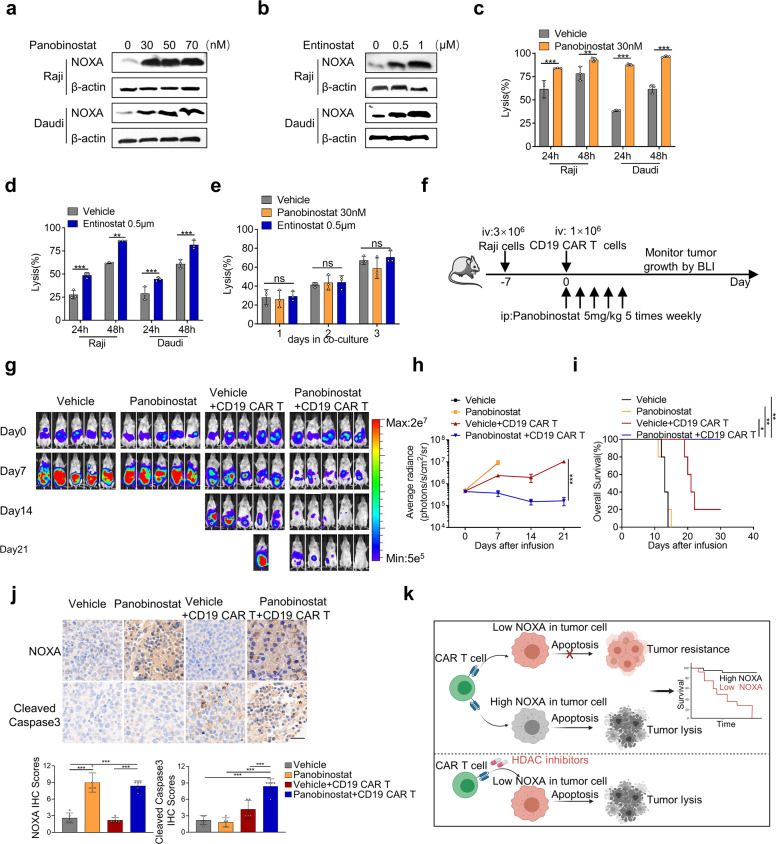
Histone deacetylase inhibitors could enhance tumor cell vulnerability to CAR T cells. **a**, **b** Western blot analysis of Raji cells and Daudi cells treated with panobinostat (**a**) or entinostat (**b**) for 24 h. **c**, **d** Cytotoxic analysis of Raji cells and Daudi cells pretreated with 30 nM panobinostat (**c**) or 0.5 μM entinostat (**d**) for 24 h and then cocultured with CD19 CAR T cells over time. **e** Cytotoxic analysis of NOXA^KO^ Nalm6 cells pretreated with 30 nM panobinostat or 0.5 μM entinostat for 24 h and then cocultured with CD19 CAR T cells over time. Values in (**a**–**e**) were shown as the mean ± SD of triplicates. **f** Experimental timeline comparing the antitumor ability of vehicle alone, panobinostat alone, the combination of vehicle and CD19 CAR T cells, and combination of panobinostat and CD19 CAR T cells in mice bearing Raji-luc xenograft tumors (*n* = 5 mice per group). **g** Raji tumor progression as evaluated by bioluminescence imaging. **h** Mouse tumor burden (average radiance). The indicated *P* value was determined by two-way ANOVA test. **i** Survival analyses of mice treated with vehicle alone, panobinostat alone, the combination of vehicle and CD19 CAR T cells, and combination of panobinostat and CD19 CAR T cells. Log-rank tests were used to analyze the significance between four groups. **j** Representative images of IHC staining for NOXA and cleaved caspase3 from Raji tumor xenografts treated with the indicated treatments. Magnification: ×40, Scale bars: 20 μm. Quantification of IHC staining using IHC scores was represented as mean ± SD of five mice per group. *P* values were calculated with the two-tailed Student’s *t* test. **k** Schematic drawing summarizing our findings. Values are shown as the mean ± SD. ns: not significant (*P* > 0.05); *P < 0.05, **P < 0.01, and ***P < 0.001

Several histone deacetylase (HDAC) inhibitors have been shown to induce cell death of hematologic malignancies by modulating NOXA expression.^33,37,38^ Based on our findings that overexpression of NOXA via gene transfer improved the susceptibility of tumor cells to CAR T cells, we attempted to investigate whether HDAC inhibitors might obtain similar biological responses by pharmacologic upregulation of NOXA. We identified that panobinostat, a pan-HDAC inhibitor, caused an increase in NOXA protein levels in Raji and Daudi cells (Fig. 6a). A class I HDAC inhibitor, entinostat, was also shown to upregulate NOXA protein levels in Raji and Daudi cells (Fig. 6b). Prior to the addition of CD19 CAR T cells, we pretreated tumor cells with 30 nM panobinostat or 0.5 μM entinostat for 24 h, both of which showed no effect on the viability of Raji and Daudi cells (Supplemental Fig. 4a–d). Notably, when tumor cells were pretreated with HDAC inhibitors, we observed a dramatically increased cytotoxic effect of CAR T cells (Fig. 6c, d). More importantly, we found that HDAC inhibitors did not increase the susceptibility of NOXA^KO^ Nalm6 cells to CAR T-cell therapy (Fig. 6e), indicating that HDAC inhibitors enhanced the therapeutic efficacy of CAR T cells dependently on NOXA expression. Finally, we explored the efficacy of the combination of HDAC inhibitors and CD19 CAR T-cell therapy in a mouse xenograft model using Raji cells (Fig. 6f). Consistent with our in vitro results, the growth of Raji tumors was significantly suppressed by the combination of panobinostat and CD19 CAR T cells as compared with either panobinostat or CD19 CAR T cells alone (Fig. 6g–i). Notably, we also observed in vivo that panobinostat in synergy with CD19 CAR T cells increased the expression of NOXA and cleaved caspase3 in the tumors of xenografted mice (Fig. 6j).

Altogether, our in vitro and in vivo results suggested that pharmacological targeting NOXA via HDAC inhibitors might be a promising strategy to overcome resistance to CAR T-cell therapy in B-cell malignancies.

## Discussion

Despite the inspiring advances in CAR T-cell therapy for hematologic tumors, some patients still do not respond to this treatment.^9,39–41^ The mechanism enabling evasion of antigen-positive tumor cells from CAR T-cell therapy is currently presumed to be CAR T-cell dysfunction.^42^ In this study, we performed an unbiased CRISPR/Cas9 screening to reveal novel tumor-intrinsic perturbations that lead to CAR T-cell therapy failure.

We identified and confirmed the key role of NOXA in CAR T-cell cytotoxicity in cancer cells through knockout and overexpression of NOXA in B-lymphoid cell lines using a variety of CAR T cells, including CD19 CAR T, CD20 CAR T, and tandem CD19/20 CAR T cells. Furthermore, we revealed that knockout of NOXA attenuated CAR T cell-mediated apoptosis of tumor cells, thereby conferring tumor cell resistance to CAR T cells. In addition to in vitro experiments, analysis involving R/R B-cell lymphoma patients treated with tandem CD19/20 CAR T-cell treatment revealed poorer response and survival when patients had low NOXA protein levels in tumor samples (Fig. 6k).

T cells kill target cells through induction of apoptosis, either via death receptors to initiate the extrinsic apoptotic pathway, or by secreting granzymes and perforin to trigger the mitochondrial-mediated intrinsic apoptotic pathway.^43^ Recently, using CRISPR/Cas9 screening, two laboratories demonstrated that impaired extrinsic apoptotic signaling in tumor cells dampened CD19 CAR T cytotoxicity and drove CAR T-cell dysfunction,^20,22^ supporting the significance of apoptosis pathway in the CAR T-cells effectiveness. Consistent with their results, we identified that extrinsic apoptotic signaling molecules, including CASP8 and FADD, were enriched in our CRISPR screening. Moreover, mitochondrial-mediated intrinsic apoptosis molecule represented by NOXA was also enriched, possibly attributed to the low effect-target ratio, long duration, and repeated CAR T-cell challenges from the coculture model used in our screening system. Our CRISPR screening system might mimic the in vivo tumor conditions and enable discovery of more candidate proteins driving tumor cells to escape CAR T-cell therapy.

NOXA protein, known as a sensitizer for inducing apoptosis, is a pro-apoptotic BCL2 family protein.^44^ Several studies have reported the significance of NOXA in regulating the cytotoxic effect of a variety of chemotherapies, BCL2 inhibitors and targeted therapies.^33,34,37,45–47^ A recent study also identified disruption of NOXA conferred resistance to natural killer cell cytotoxicity by a CRISPR screening in SUDHL4 cells, a B-cell lymphoma cell line, implying that NOXA might be a critical regulator of the antitumor response of cancer cells to distinct modalities of therapies.^48^ Future work needs to further explore whether patients with high expression of NOXA would respond better to a range of immune-based therapies given its important role in regulating apoptosis.

Resistance to apoptosis is a hallmark of tumor biology, often conveyed by halted apoptosis machinery.^49^ Hence, targeting apoptotic machinery of tumor cells provides novel opportunities to sensitize the tumor cells to multiple therapeutic options.^50^ Numerous previous studies have reported that NOXA expression was dynamically regulated at the epigenetic and chromatin levels.^51^ HDAC inhibitors have been repeatedly shown to induce NOXA expression in lymphoma cells in preclinical studies.^33,52–54^ Our in vitro and in vivo study confirmed that HDAC inhibitors (panobinostat and entinostat) dramatically increased the cytotoxic efficacy of CAR T-cell therapy. To date, four HDAC inhibitors including panobinostat, a pan-deacetylase inhibitor, have been approved by the FDA for treating hematologic tumors in clinic.^55,56^ Entinostat is a highly selective class 1 HDAC inhibitor, has exhibited synergy in preclinical models when combined with rituximab for treating B-cell lymphoma.^57^ In addition to panobinostat and entinostat, other HDAC inhibitors, such as kendine 92 and SAHA have also shown NOXA protein increases in chronic lymphocytic leukemia cells, which may be used as reference drugs in combination with CAR T-cell therapy in hematologic cancers.^58^ Our exciting in vitro and in vivo results, although requiring further exploration, may provide a strong mechanistic rationale for the incorporation of HDAC inhibitors into CAR T-cell-based therapy in B-cell malignancies.

In summary, our genome-scale CRISPR/Cas9 screening identified a novel mechanism of resistance to CAR T cells, potentially revealing that NOXA might be a good marker for predicting the susceptibility of CAR T-cell therapy. In addition, pharmacologic induction of NOXA would be a promising strategy to enhance sensitivity to CAR T-cell therapy in B-cell malignancies.

## Materials and methods

### Cell lines and cell culture

Nalm6, Raji, and Daudi cells were purchased from ATCC (Manassas, VA). Their identities were verified by short tandem repeat DNA analysis. These cells were infected with the lentivirus pRRLSIN-EF-1α-GFP-luc to express GFP virus and firefly luciferase. Nalm6 cells were infected with a pRRLSIN-EF-1α-mCherry-luc virus to express mCherry and firefly luciferase. Infected cells were sorted based on GFP or mCherry expression by flow cytometry. All the above cells were tested for mycoplasma contamination before the experiment. All cell lines were cultured in RPMI-1640 medium (Gibco, USA) supplemented with 10% fetal bovine serum (Gibco) and 100 U/mL penicillin/streptomycin (Gibco).

### CAR T-cell manufacturing

All CAR T designs used in this study have been described in our previous reports.^16,19^ Different CAR constructs in this study are displayed in supplemental Fig. 5a, b. The CAR constructs were synthesized and cloned into the lentivirus PRRLSIN-EF-1α-GFP vector. pRRLSIN and PSPAX2 and PMD2G packaging plasmids were transfected into 293T cells. Lentiviral supernatants were harvested 48 h later and stored at −80 °C. Peripheral blood mononuclear cells (PBMCs) were derived from healthy donors. One microgram/mL anti-CD3 monoclonal antibody (OKT3, Takara, Japan) and RetroNectin (Takara) were precoated overnight at 4 °C to activate T cells. Primary T cells were cultured in X-VIVO 15 medium (Lonza, Switzerland) containing 10% fetal bovine serum (Gibco) and 300 U/ml recombinant human IL-2 (PeproTech, USA). After 2 days, the virus supernatant was added to RetroNectin-coated plates and centrifuged for 2 h at 2000×*g*. Activated T cells and 4 μg/mL polybrene (Sigma–Aldrich, USA) were added to the plates and centrifuged at 1000×*g* for 10 min. After 24 h, CAR T cells were replaced with fresh medium to reduce the toxicity of polybrene. CAR T cells were maintained in X-VIVO 15 medium containing 300 U/ml recombinant human IL-2.

### Genome-wide CRISPR/Cas9 knockout library screening

The Brunello sgRNA library consisting of 77441 sgRNAs targeting 19110 protein-coding genes (4 sgRNAs per gene) was used to transduce the target-cell line Nalm6. Cells transduced with the lentivirus were selected under puromycin pressure. Day 0 cells containing the library were collected as the control group. CD19 CAR T cells were cocultured with transduced Nalm6 cells at an effector-to-target (E:T) of 1:50, and CD19 CAR T cells were added at an E:T of 1:50 every 3 days. After 15 days, the remaining living Nalm6 cells were harvested for genomic DNA extraction. The sgRNA sequences were amplified by NEBNext^®^ High-Fidelity 2X PCR Master Mix and subjected to massive parallel amplicon sequencing. The MAGeCK v0.5.7 algorithm was used to analyze the sgRNA read counts. Candidate genes were identified based on log2 fold change and *P* value for all sgRNAs. To identify significant pathways enriched in the CRISPR screening, hypergeometric distribution statistics were used to compute overlapping gene sets with the top enriched genes (log2 fold change>2 and *P* < 0.05).

### Establishment of NOXA knockout and overexpression cell lines

#### NOXA knockout

Two sgRNA sequences targeting NOXA were cloned into a lentiCRISPRv2-GFP vector and confirmed by sequencing. The sgRNA sequences targeting NOXA were extracted from the CRISPR Brunello library. Nalm6 cells were infected with sgNOXA or control sgRNA lentivirus supernatants. Infected cells were sorted based on GFP by flow cytometry. All the sgNOXA sequences were as follows: sgNOXA-1: TTCTTGCGCGCCTTCTTCCC; sgNOXA-2: TTTGCTTTCCTTCTCAGAGC.

#### NOXA overexpression

The NOXA construct was synthesized and cloned into the lentivirus pRRLSIN-EF-1α-GFP vector. Raji cells were infected with NOXA or empty vector lentivirus supernatants. Infected cells were sorted based on GFP by flow cytometry.

#### Drugs

Panobinostat and entinostat were obtained from Selleck (USA) and APE×BIO (USA), respectively.

#### Growth competition assay

NOXA^KO^ (GFP^+^) or NOXA^OE^ cells (GFP^+^) and control tumor cells (mCherry^+^) were mixed at a 1:1 ratio with control or CAR T cells. The change in the GFP^+^/ (GFP^+^ + mCherry^+^) ratio in the mixed cells was detected by flow cytometry over time.

#### Clinical tissue specimens and Ethics statement

Tumor tissue samples and patient information were obtained from a clinical trial of tandem CD19/20 CART-cell therapy in R/R B-cell lymphoma (NCT03097770),^16,35^ which was approved by the Ethics Committee of Chinese PLA General Hospital and conducted in accordance with principles of the Declaration of Helsinki. All patients provided informed consent and authorization.

#### Immunohistochemistry and immunohistochemical scoring

The samples were immunostained as described previously.^16^ NOXA and cleaved caspase3 staining were localized to the cytoplasm of tumor cells and scored according to intensity (0/1/2/3) and positive rate (grouped into quartiles (0–4)). The intensity and positive scores were multiplied to form an IHC score. Low expression scores ranged from 0 to 4, and the scores that exceeded 4 were identified as high expression.^36^

Additional methods information can be found in Supplementary Materials.

### Statistical analysis

Data were analyzed and visualized using GraphPad Prism 6 (GraphPad Software Inc., La Jolla, CA, USA). Two-tailed Student’s *t* or two-way ANOVA test was used to compare the significant differences between the relevant groups. The area under the receiver operating characteristic (ROC) curve was calculated to evaluate the sensitivity of NOXA IHC scores in predicting response to CAR T-cell therapy. Survival analysis was analyzed using the log-rank test. Significant significance among groups was defined as *P < 0.05; **P < 0.01; ***P < 0.001.

## Supplementary information

Supplemental Materials

Dataset 1

## Data Availability

All data that support the findings of this study are available to the researchers on reasonable request.
