# Supplementary material for: Identification of NOXA as a pivotal regulator of resistance to CAR T-cell therapy in B-cell malignancies
Source: Signal Transduct Target Ther. 2022 Apr 4;7:98. doi: 10.1038/s41392-022-00915-1 (PMC8977349; doi:10.1038/s41392-022-00915-1)
Supplement: Supplementary file 1 — Supplemental Materials [file 41392_2022_915_MOESM1_ESM.docx]

Supplemental Materials for

**Identification of NOXA as** **a pivotal regulator** **of resistance to CAR T-cell therapy in B-cell malignancies**

Xin Yan^1, 2†^, Deyun Chen^2†^, Yao Wang^2^, Yelei Guo^2^, Chuan Tong^2^, Jianshu Wei^2^, Yajing Zhang^2^, Zhiqiang Wu^2^*, Weidong Han^1, 2,3^*

^1^School of Medicine, Nankai University, Tianjin, China

^2^Department of Bio-therapeutic, the First Medical Center, Chinese PLA General Hospital, Beijing, China

^3^National Clinical Research Center for Hematologic Diseases, the First Affiliated Hospital of Soochow University，Suzhou, China

^†^These authors contributed equally: Xin Yan and Deyun Chen

*Correspondence to: Zhiqiang Wu ([wuzhiqiang1006@163.com](mailto:wuzhiqiang1006@163.com)) and Weidong Han ([hanwdrsw69@yahoo.com](mailto:hanwdrsw69@yahoo.com))

**This PDF file includes:**

Supplemental Materials and Methods

Supplementary Figures.1 to 5

**Other Supplementary Materials for this manuscript include the following:**

Supplementary Table 1

**Materials and Methods**

RNA sequencing and data analysis

sgCONT (nontargeting sgRNA) and NOXA^KO^ Nalm6 cells were treated with CD19 CAR T cells at an E:T of 1:10 in triplicate and isolated by fluorescent cell sorting after 24 h. RNA extraction, library building and sequencing, and data analysis were all performed by The Beijing Genomics Institute. Differentially expressed genes were defined as genes with *P* values <0.05 and log_2_FC>1. Pathway enrichment analysis of differentially expressed genes was implemented by the hypergeometric overlap statistic tool (Metascape)^1^.

Western blot

sgCONT and NOXA^KO^ Nalm6 cells were treated with CD19 CAR T cells at an E:T of 1:10 in triplicate and isolated by fluorescent cell sorting after 24 h. The tumor cells in the different conditions were collected, and the total proteins were extracted and separated by 15% or 12% precast Bis-Tris gels (Genscript, China), and then Western blot assays were performed. The detection of Bax protein requires separation of mitochondrial and cytoplasmic components, using a mitochondrial isolation kit (Beyotime Institute of Biotechnology), according to the manufacturer's protocol. Antibodies used for Western blotting were as follows: NOXA (Cell Signaling, USA , 1:1000), Caspase3 (Cell Signaling, 1:1000), Cleaved Caspase3 (Cell Signaling, 1:1000), Caspase9 (abcam, UK, 1:1000), Cleaved Caspase9 (Cell Signaling, 1:1000), Cleaved PARP (Cell Signaling, 1:1000), Bax(Cell Signaling, 1:1000), HSP(Cell Signaling, 1:1000), β-actin (ZSGB-BIO, China, 1:1000), anti-rabbit HRP-conjugated antibody (Cell Signaling, 1:2000), PARPanti-mouse HRP-conjugated antibodies (Cell Signaling, 1:3000).

Cytotoxicity assay

CAR T and target cells were cocultured in 96-well plates with different E:T ratios. Forty microliters of 2× D-fluorescein solution (300 μg/ml) was added to each well, and the luciferase signal was detected by Varioskan™ LUX (Thermo Fisher, USA) after 2-5 minutes. The percentage of lysis was calculated using the following formula: 1-([sample value]-[negative control])/ ([positive control]-[negative control]).

Flow cytometry analysis.

All operations were carried out using the protocol provided by the manufacturer. All samples were detected using multicolor flow cytometry on a DxFLEX flow cytometer (BECKMAN COULTER), and data were analyzed using FlowJo software v.10 (FlowJo, LLC, Ashland, OR, USA). The antibodies used in the study were as follows: biotin-SP-AffiniPure F(ab)’2 fragment (Jackson ImmunoResearch, USA), PE Streptavidin (BD Biosciences, USA), CD45RO (Biolegend, USA), CD62L (Biolegend), Ki67 (Biolegend), CD19 (Biolegend), CD25 (Biolegend), CD69 (Biolegend) and CD3 (Biolegend).

For observation of apoptosis of tumor cells, CAR T cells and tumor cells were cocultured at an E:T ratio of 1:10 for 24 h, and an apoptosis assay was conducted with Annexin V/7-ADD using the protocol provided with the apoptosis detection kit (BD Biosciences). A mitochondrial membrane potential assay was performed with a JC-1 probe kit (Beyotime, China).

For observation of memory and activated phenotype of CAR T cells upon Nalm6 cells stimulation, CAR T and target cells were cocultured at an E:T ratio of 1:10 for 24 h, and then the cells were stained with the corresponding antibodies for flow cytometry analysis.

Cell viability and proliferation assay

Cells were cultured in 96-well plates and added to the indicated drugs. A CCK-8 assay was conducted after incubation for 24 h and 48 h. Cells supplemented with vehicle were defined as having 100% cell viability. The absorbance (optical density, OD) was read at 450 nm by an enzyme-linked immunosorbent assay (ELISA) plate reader. Cell viability was calculated as follows: (OD treatment/OD control) ×100%.

For observation of in vitro proliferation, 1 × 10^5^ cells were cultured in 12-well plates for 9 days, 1640 medium was supplemented every 3 days, and the living cells were counted with trypan blue staining.

Quantitative real-time PCR (qPCR)

sgCONT (nontargeting sgRNA) and NOXA^KO^(targeting sgNOXA-1) Nalm6 cells were treated with CD19 CAR T cells at an E:T of 1:10 in triplicate and isolated by fluorescent cell sorting after 24 h. Total RNA was extracted using RNeasy Plus Mini Kit(Qiagen, Germany). cDNA was synthesized using a reverse transcription kit (Thermo Fisher Scientific, USA). q-PCR was carried out with SYBR Green master mix (TAKARA) on StepOnePlus Real-Time PCR system. The gene expression was normalized to β-ACTIN RNA. Relative expression levels were analyzed by the 2^-ΔΔCT^ method. Samples were run in triplicate. Sequences of primers are listed in the table below.

| BAX | Forward | ACCAAGAAGCTGAGCGAGT |
| --- | --- | --- |
|  | Reverse | ATGGTCACGGTCCAACCAC |
| HRK | Forward | CTCCCCACCTACTGGCCTT |
|  | Reverse | CAAGTTCCGCCTGCCGAG |
| CDK6 | Forward | ACAGAGCACCCGAAGTCTTG |
|  | Reverse | CCTGGGAGTCCAATCACGTC |
| CDK14 | Forward | GAGCAAAATCCGTCCCTAGC |
|  | Reverse | TGGTGTTCCAAGAACCAGAAAT |
| BCL2 | Forward | CTTCGCCGAGATGTCCAGC |
|  | Reverse | CGAACTCAAAGAAGGCCACAAT |
| β-Actin | Forward | GGCACCCAGCACAATGAAG |
|  | Reverse | CCGATCCACACGGAGTACTTG |

Mouse study

4-6 week–old female NOD-Prkdcscid-Il2rgnull mice (NPG/Vst, VITALSTAR) were used for in vivo studies. For our intravenous xenograft mouse model, mice were intravenously injected with 1×10^5^ sgCONT or NOXA^KO^(targeting sgNOXA-1) Nalm6-luc cells in 100ul PBS. Purified CD19 CAR T cells were sorted using magnetic beads (Miltenyi Biotec) at 3 days post-lentivirus infection. After 7 days of tumor growth,1 × 10^6^ CD19 CAR T or control T cells in 100ul PBS were injected into mice via tail vein

For our intraperitoneal xenograft model, we injected 3 × 10^6^ Raji-luc cells in a Matrigel (Invitrogen) mixture into mice intraperitoneally. 7 days after tumor growth, mice were randomly divided into four groups: intraperitoneal injection of vehicle alone, panobinostat alone, intravenous infusion of sorted CD19 CAR T cells alone, or panobinostat combined with CAR T cells. The dose of sorted CD19 CAR T cells was 1×10^6^ for each mouse intravenously. Panobinostat or vehicle was dosed at 5 mg/kg i.p. daily, five times weekly for up to 3 week.

n = 5 mice per group. Tumor burdens were monitored and quantified by Bioluminescence imaging (BLI) on IVIS Imaging System (PerkinElmer). BLI signals were shown as the average flux (photons per second∕area[mm^2^]) and acquired by Living Image software (PerkinElmer).

**
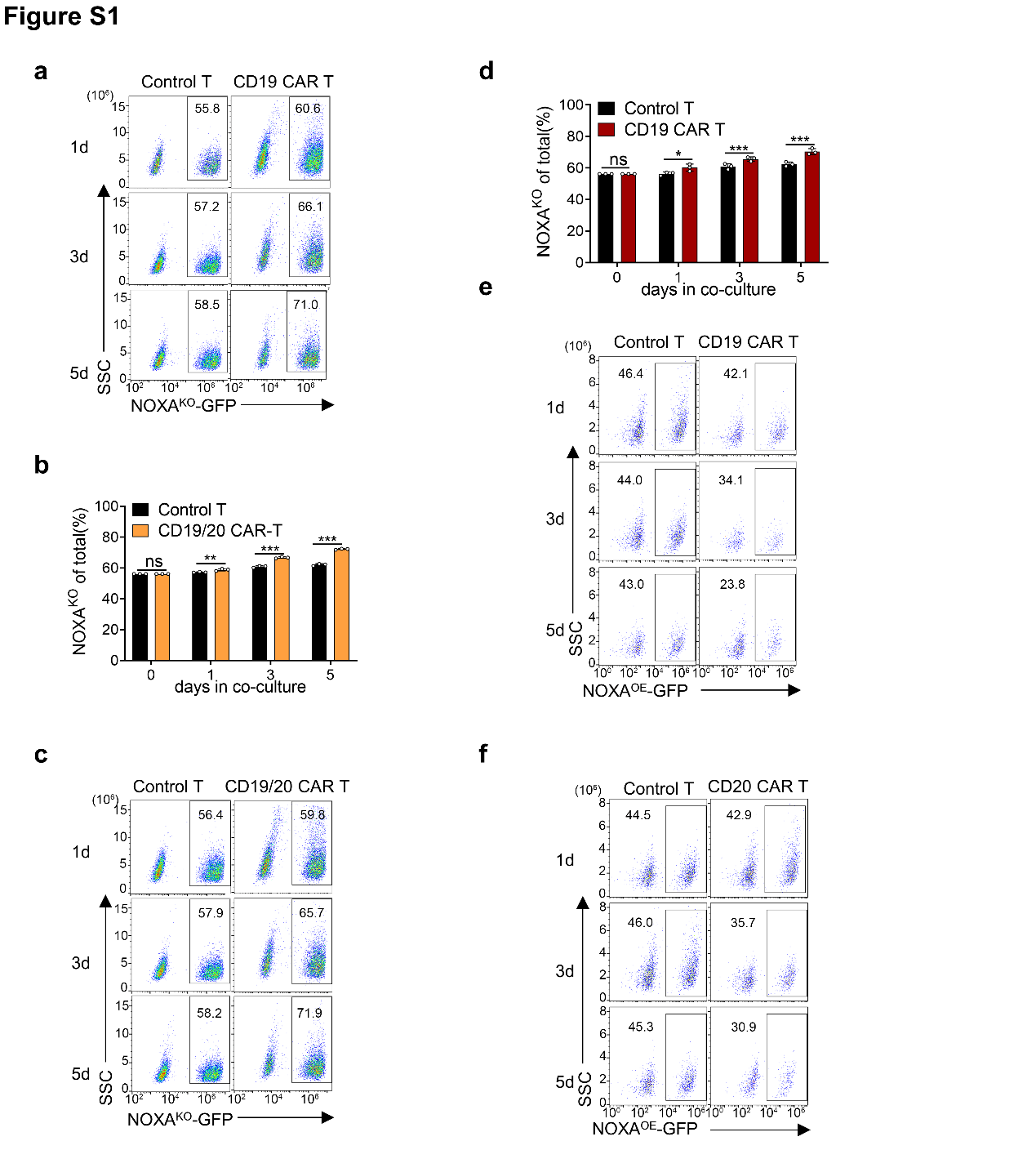
**

**Figure S1: NOXA plays a pivotal role in resistance to CAR T cells**

**a, b** Representative flow cytometry plots of NOXA^KO^ Nalm6 cells in the presence of CD19 CAR T cells at an E:T ratio of 1:10 **(a)** or the proportion of NOXA^KO^ Nalm6 cells at an E:T ratio of 1:20 **(b)** over time are shown. **c, d** Representative flow cytometry plots of NOXA^KO^ Nalm6 cells in the presence of tandem CD19/20 CAR T cells at an E:T ratio of 1:10 **(c)** or the proportion of NOXA^KO^ Nalm6 cells at an E:T ratio of 1:20 **(d)** over time are shown.

**e, f** Representative flow cytometry plots of NOXA^OE^ Raji cells in the presence CD19 CAR T cells **(e)** and tandem CD19/20 CAR T cells **(f)**.The values on the flow cytometry plots in **(a)** and **(c)** refered to the percentage of NOXA^KO^ Nalm6 cells in total Nalm6 cells. The values on the flow cytometry plots in **(e)** and **(f)** refered to the percentage of NOXA^OE^ Raji cells in total Raji cells. Differences among groups were calculated with two-way ANOVA test. Values were shown as the mean ± SD of triplicates. **P* < 0.05, ***P* < 0.01, and ****P* < 0.001.

**
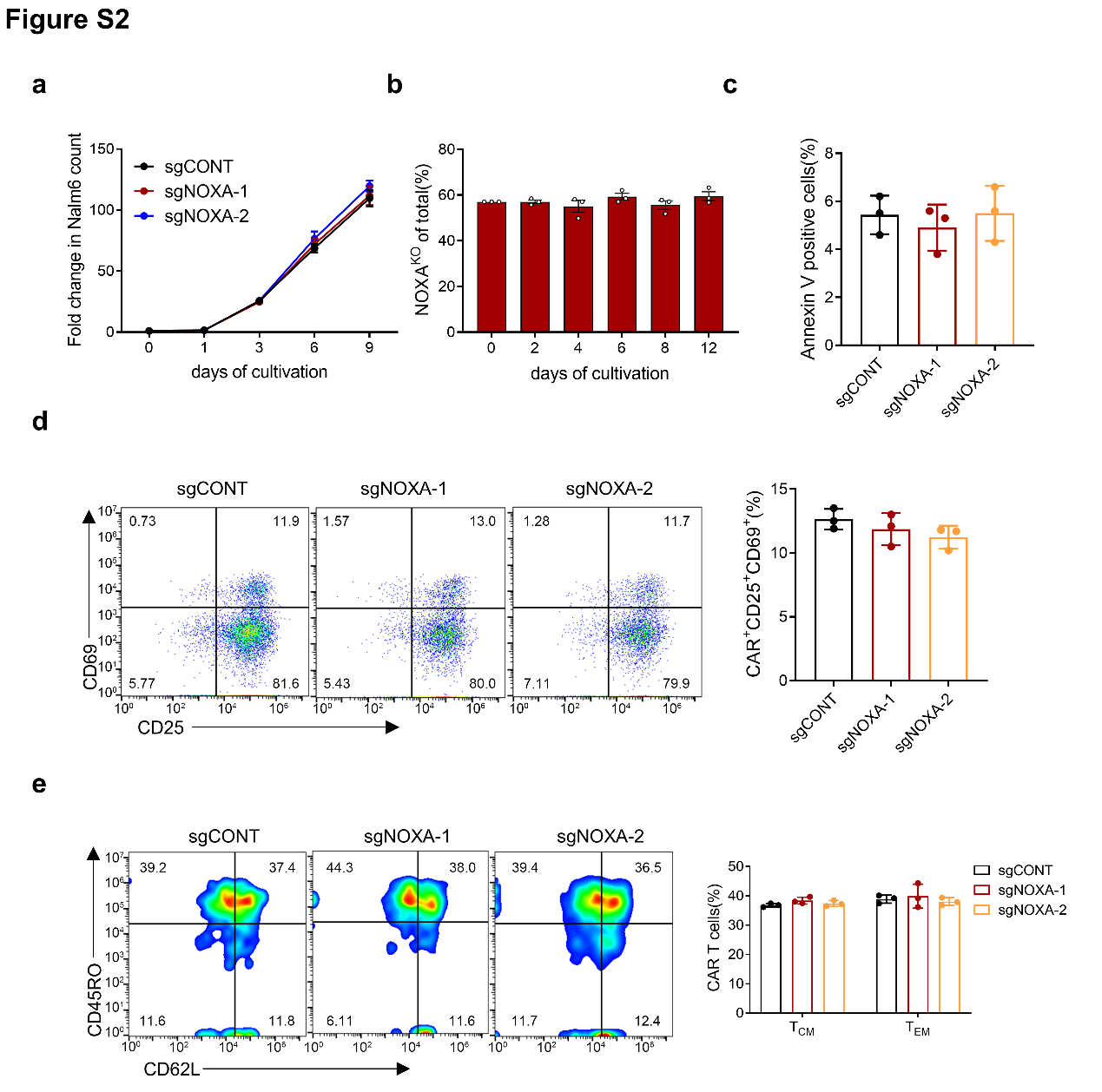
**

**Figure S2: Effect of NOXA loss on tumor cell proliferation and apoptosis and on the phenotype of co-cultured CAR T cells**

**a** Effect of knockout NOXA on tumor cell growth. **b** mCherry^+^ sgCONT Nalm6 cells were combined with GFP^+^ NOXA^KO^ Nalm6 cells at an approximately 1:1 ratio. Proportion of GFP^+^ tumor cells over time is shown. **c** Effect of NOXA knockout on tumor cell apoptosis (Annexin V^+^) after 7 days. **d** Flow cytometry analysis of CD25^+^CD69^+^ central memory T cells after 24 h of coculture with Nalm6 cells. **e** Flow cytometry analysis of CD62L^+^CD45RO^+^ central memory T cells after 24 h of coculture with Nalm6 cells. Differences among groups were calculated with one-way ANOVA tests. Values were shown as the mean ± SD of triplicates.

**
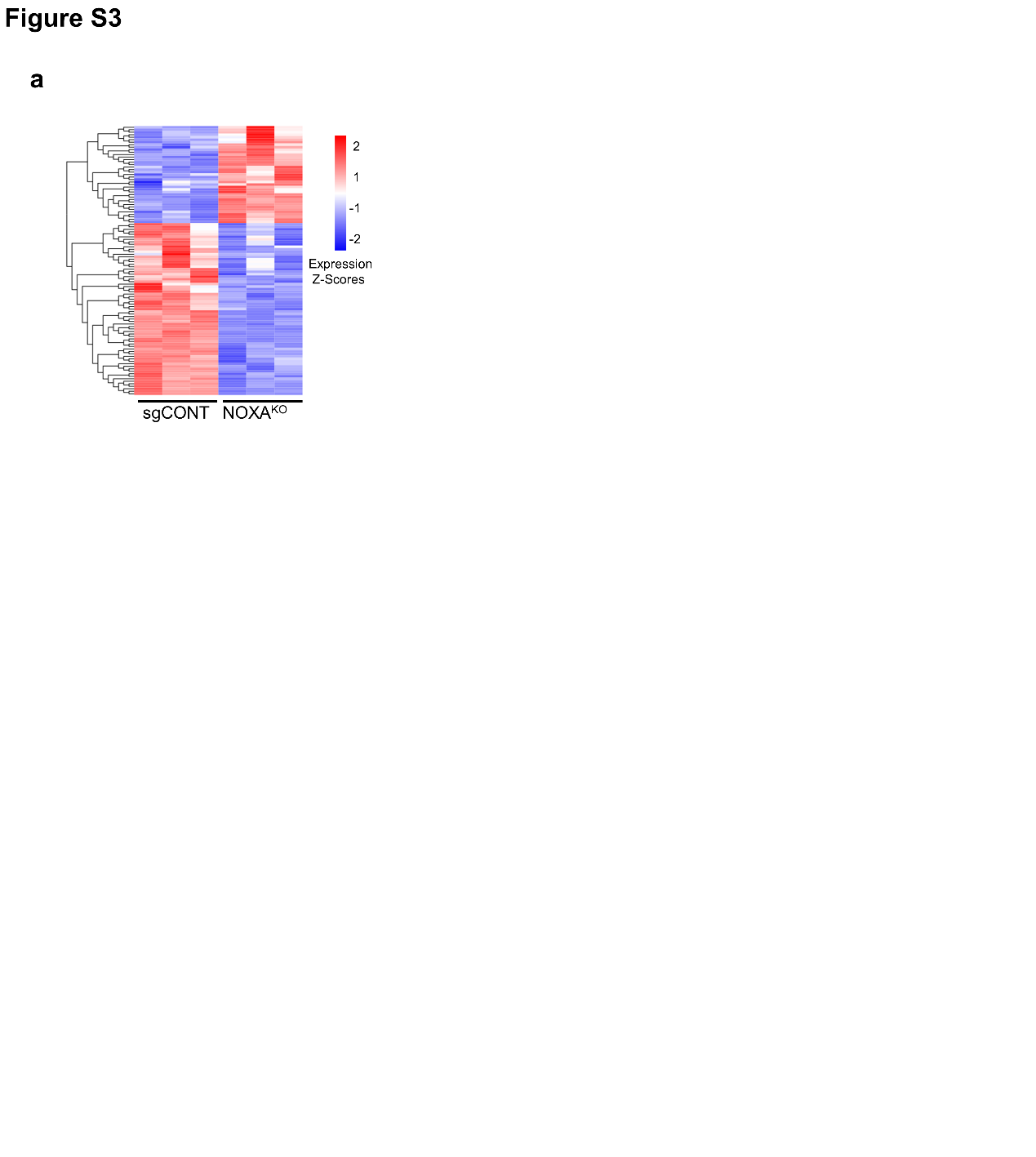
**

**Figure S3: Hierarchical clustering of the RNA sequencing data**

**a** Hierarchical clustering of the RNA sequencing data showed differentially expressed genes between sgCONT and NOXA^KO^ Nalm6 cells after coculture with CD19 CAR T cells

**
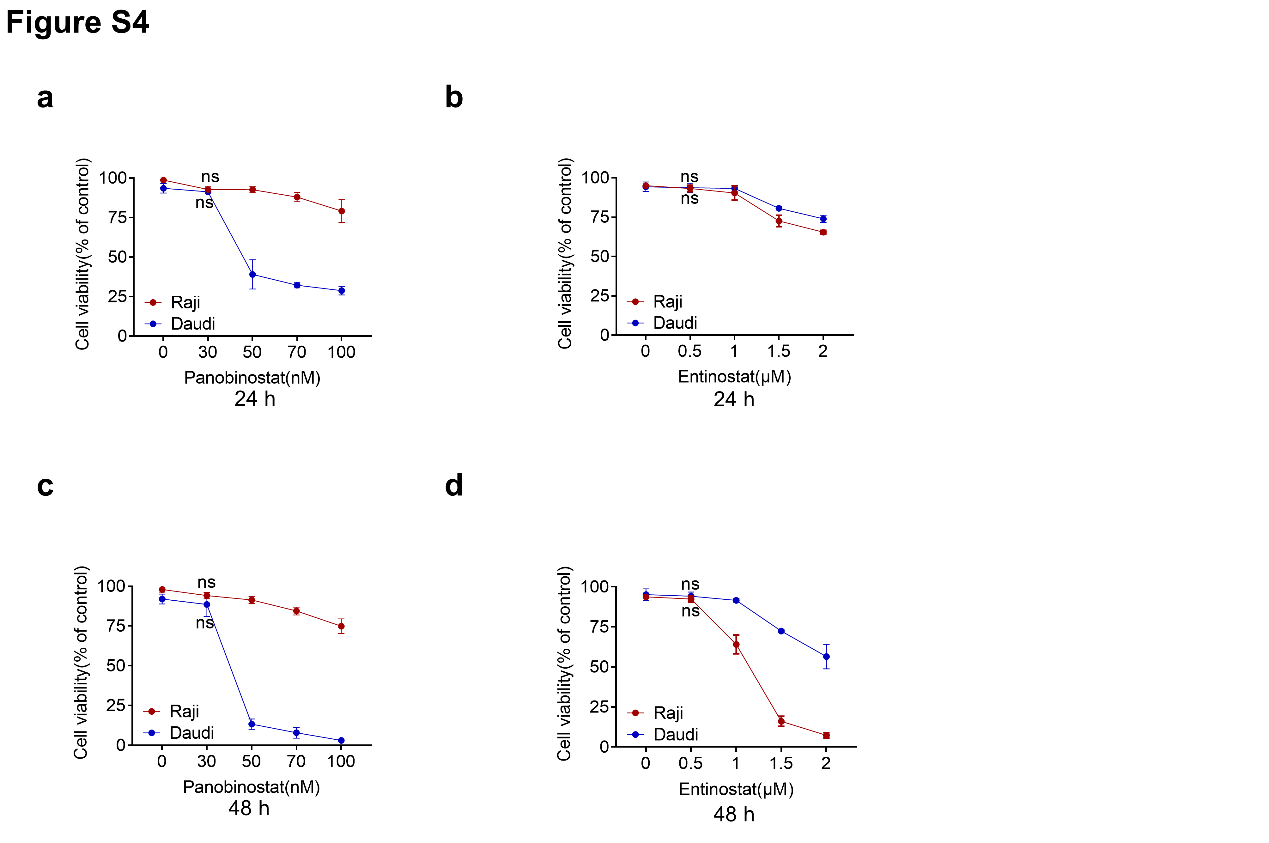
**

**Figure S4: Cytotoxicity assay of panobinostat and entinostat for Raji cells and Daudi cells**

**a-d** Raji cells and Daudi cells were treated with panobinostat **(a)** or entinostat **(b)** for 24 h. Raji cells and Daudi cells were treated with panobinostat **(c)** or entinostat **(d)** for 48 h. Cell viability was determined with a CCK-8 assay. Values were shown as the mean ± SD. ns: not significant (*P* > 0.05)

.

**
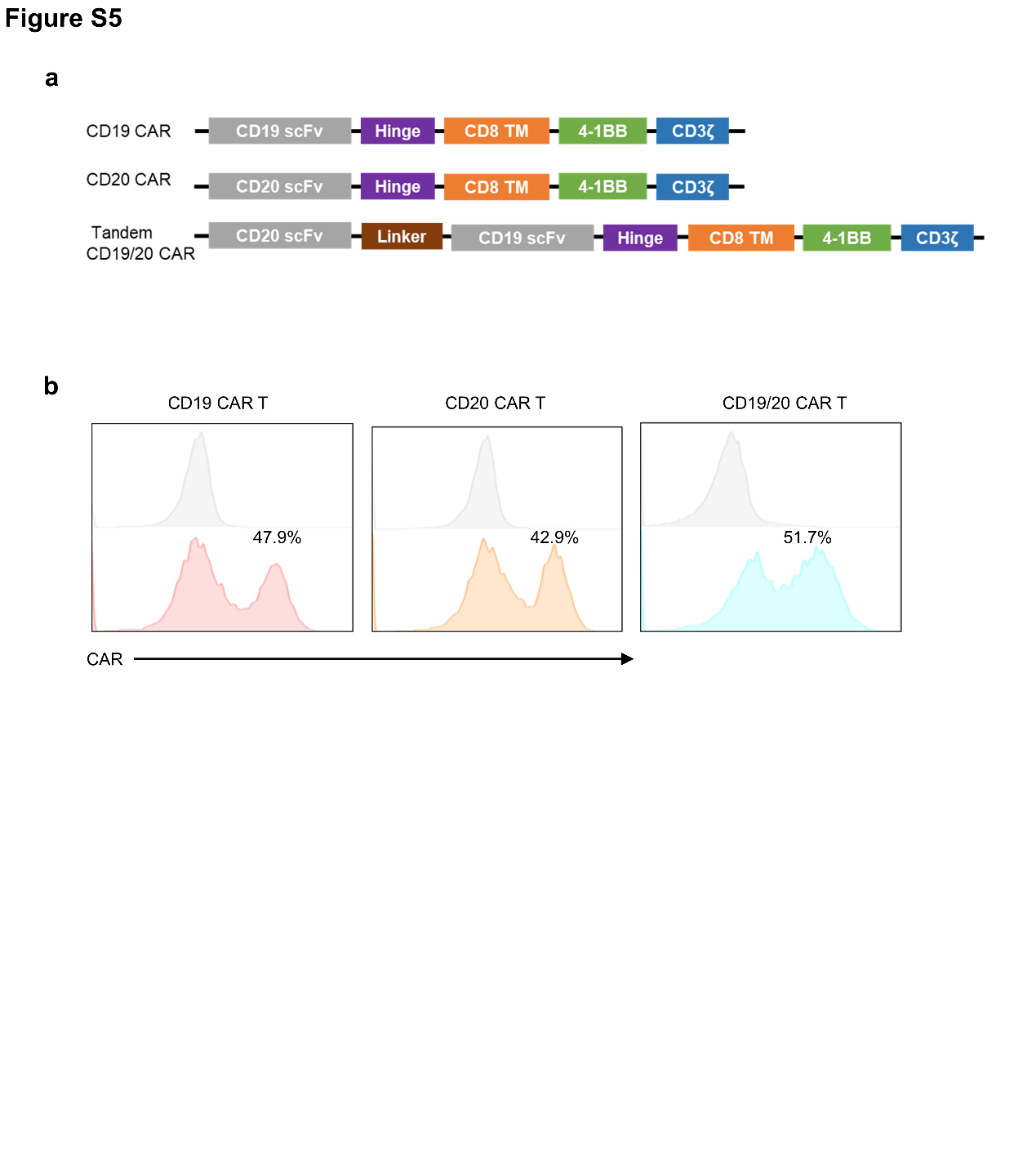
**

**Figure S5: CAR constructs and** **efficiency of CAR transduction**

**a** Schematic representation of the different CAR constructs used in this study. **b** CAR expression determined by labelling of CAR T cells with anti-CAR or anti-idiotype antibody 3 days after transduction and analysis by flow cytometry.

**Supplemental Table S1**.**Complete list of candidate genes in the CRISPR screen.**

lfc: log_2_FC;score: RRA score; rank: rank by RRA score

**References**

1. Zhou, Y. et al. Metascape provides a biologist-oriented resource for the analysis of systems-level datasets. *Nat. Commun.* **10**, 1523 (2019).
